# Advances in Targeted Therapy for Human Epidermal Growth Factor Receptor 2-Low Tumors: From Trastuzumab to Antibody-Drug Conjugates

**DOI:** 10.14740/wjon2718

**Published:** 2026-03-05

**Authors:** Zhi Nuo Zheng, Xiao Han Ye, Xiao Ban Shen, Tao Li, Jie Guo

**Affiliations:** a Health Science Center, Ningbo University, Ningbo, China; b HEALTH BioMed Research and Development Center, Health BioMed Co., Ltd., Ningbo, Zhejiang, China; c School of Materials Science and Chemical Engineering, Ningbo University, Ningbo, China

**Keywords:** Human epidermal growth factor receptor 2, Trastuzumab, HER2-low tumors, Antibody-drug coupling, Breast cancer, Gastric cancer

## Abstract

The assessment of human epidermal growth factor receptor 2 (HER2) expression status has evolved from the traditional binary classification of positive/negative to a continuum that includes HER2-low expression. This shift has redefined the treatment landscape for approximately half of breast cancer patients. Trastuzumab, the cornerstone monoclonal antibody targeting HER2, significantly improves outcomes in HER2-high patients by blocking downstream signaling pathways and mediating antibody-dependent cellular cytotoxicity. However, its efficacy remains limited in tumors with low HER2 expression. In recent years, the emergence of antibody-drug conjugates (ADCs) has overcome this limitation. Represented by trastuzumab deruxtecan (T-DXd), a new generation of ADCs has successfully extended therapeutic benefits to HER2-low tumors through high drug-to-antibody ratios, cleavable linkers, and potent bystander effects. T-DXd has been established as the new standard of care for previously treated patients. This review systematically outlines the evolution of HER2 expression profiles, the mechanism of action and limitations of trastuzumab, and focuses on analyzing the breakthrough role of ADCs centered on trastuzumab emtansine (T-DM1) and T-DXd in HER2-low tumors, key clinical evidence, and adverse reaction management. Additionally, it explores the application prospects of combination strategies involving ADCs with chemotherapy and immunotherapy. Finally, the article summarizes challenges facing the current treatment paradigm and outlines future directions for standardized testing and novel therapeutic development.

## Introduction

In breast and gastric cancers, the proportion of human epidermal growth factor receptor 2 (HER2)-positive patients is about 15–20% [1–3]. HER2-positive status is one of the key therapeutic targets in breast and gastric cancers with well-defined clinical and pathological features [2, 4]. The therapeutic agents targeting HER2 include monoclonal antibodies (mAbs), tyrosine kinase inhibitors (TKIs), and antibody-drug conjugates (ADCs). Over the past two decades, the treatment of patients with HER2-positive tumors has significantly improved [5]. However, the efficacy of trastuzumab on HER2-negative tumors is limited. HER2 overexpression is an important therapeutic target, but relevant studies are still scarce [6, 7]. With the development of cancer mechanisms and drug research, HER2-low tumors have attracted more and more attention, and some effect drugs for the treatment of HER2-low expression cancers have been developed. For example, the ADC form of trastuzumab and the combination administration of trastuzumab have promoted the application of trastuzumab in HER2-low tumors.

Trastuzumab is a genetically engineered human-like mAb developed through recombinant DNA techniques. Its mechanism of action involves selective binding to HER2, a receptor belonging to the tyrosine kinase family. Activation of HER2 begins with the formation of heterodimers, which triggers the phosphorylation of tyrosine kinases, ultimately leading to the initiation of several key signaling pathways, mainly including the Ras/MAPK, PI3K/AKT/mTOR, PLCγ/PKC, and JAK2/STAT pathways. These signaling pathways are vital for governing fundamental biological functions, such as cell growth, apoptosis, differentiation, angiogenesis, and cell invasiveness [8, 9]. Trastuzumab inhibits the activation of these pathways by binding to HER2, thereby achieving an inhibitory effect on tumor cells. Trastuzumab has a significant effect on HER2-positive cancers [10]. However, the development of ADC form of trastuzumab has played a significant role in the treatment of HER2-low tumors.

## HER Family and HER2

**Figure 1:**
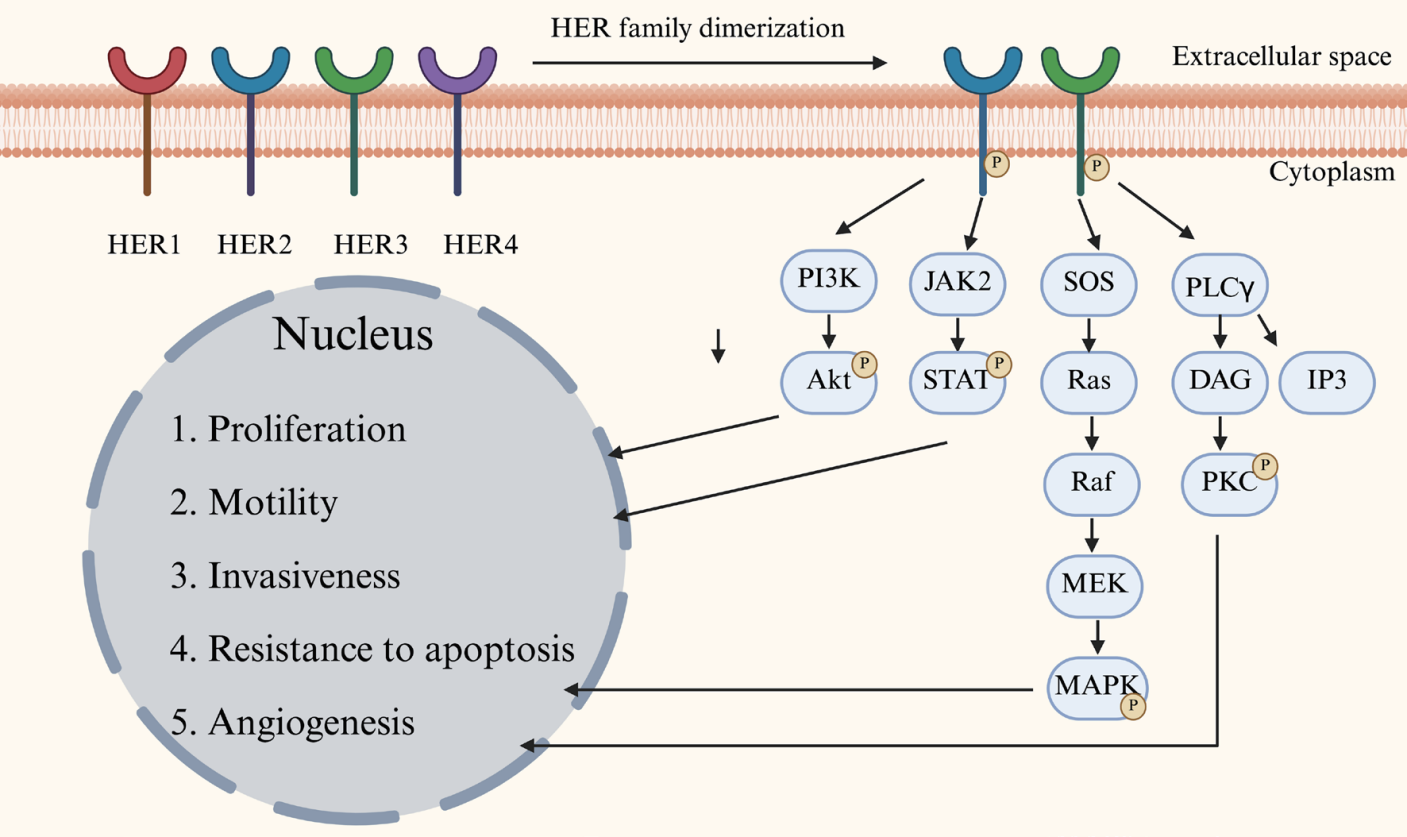
Major signaling pathways of human epidermal growth factor receptor (HER) family. There are four HER family members, HER1 to HER4. The dimerization and phosphorylation of HER activate several signaling pathways and then regulate cell activities.

The human epidermal growth factor receptor (HER) family of tyrosine kinases consists of four members, HER1 to HER4 (also known as ErbB1 to ErbB4). The trigger of downstream signaling cascades through homo- or heterodimerization is crucial in the regulation of physiological processes such as cell proliferation and differentiation. Their aberrant activation is a central mechanism driving the development of many tumors [11]. HER2 proteins, as the core dimerization chaperones of the HER family, form heterodimers (especially the HER2-HER3 complex) that exhibit significantly stronger signaling efficacy than other receptor combinations. The key mechanism of activation of this receptor is that when ligands (e.g., neuromodulators) bind to members of the HER3/epidermal growth factor receptor (EGFR) family, they induce a conformational change in the receptor, which promotes the formation of a heterodimer of HER2 and the ligand-bound receptor. This triggers autophosphorylation of the structural domains of the cytosolic tyrosine kinase, ultimately activating downstream signaling cascades [12, 13]. These cascades lead to the activation of several signaling pathways. The activated factors enter the nucleus and regulate gene expression, finally initiating various downstream cellular functions, such as promotion of cell proliferation, cell migration, cell invasion, and pericellular vascular formation, and resistance to apoptosis (Fig. 1).

HER2 is a transmembrane tyrosine kinase receptor encoded by the *ERBB2* gene, which is localized in 17q12. Overexpression of this receptor, a proto-oncogene product, can drive tumor development through aberrant signaling [14]. Traditionally, HER2 situation in tissues has been classified as positive and negative based on its protein expression level and gene amplification status. HER2 positivity means that there is too much HER2 protein on the surface of cancer cells and can lead to a more aggressive tumor, a faster growth rate, and an increased risk of recurrence and metastasis.

HER2 is an important site for the targeting of trastuzumab. Trastuzumab has a significant effect on HER2-positive conditions, but its effect on HER2-negative conditions is unsatisfactory. As a result, HER2-negative cancers are far less well known and studied than HER2-positive cancers. However, in recent years, the understanding of HER2 expression status has evolved from a traditional dichotomous model (positive/negative) to a continuous spectrum encompassing categories such as HER2-positive, HER2-low, and HER2-ultra-low. This shift primarily stems from the 2021 the American Society of Clinical Oncology (ASCO)/College of American Pathologists (CAP) guideline update, which formally introduced the classifications of HER2-low—defined as immunohistochemistry (IHC) 1+ or 2+/*in situ* hybridization (ISH) negative—and HER2-ultra-low (IHC 0 with incomplete faint staining). This allows for a more nuanced reflection of the biological continuum and clinical heterogeneity of HER2 expression [15–18]. HER2-low tumors exhibit some HER2 protein expression below conventional positivity thresholds, with molecular characteristics intermediate between HER2-negative and HER2-positive tumors. These tumors may respond to novel ADCs (e.g., trastuzumab deruxtecan (T-DXd)) [19, 20]. The establishment of this spectrum perspective emphasizes HER2 expression as a dynamic continuum, driving the development of personalized treatment strategies and advancing research into novel therapies targeting low expression levels.

According to the results of IHC and fluorescence *in situ* hybridization (FISH), HER2 status is divided into four subtypes (Table 1), which are HER2- positive, HER2-low, HER2-ultra-low and HER2-negative [21, 22]. Besides, to confirm HER2-positive type, silver staining *in situ* hybridization (SISH) or next-generation sequencing (NGS) may be used.

**Table 1:** The Definition and Testing Criteria of Four Types of HER2 Status

Type	Definition	Testing standards	
		IHC	FISH
HER2-positive	Overexpression or gene amplification of HER2 protein on the surface of tumor cells.	3+: strong and intact cell membrane staining (> 10% tumor cells)	HER2/CEP17 ratio ≥ 2.0, or *HER2* gene copy number ≥ 6.0/cell
HER2-negative	Tumor cells do not have HER2 overexpression or gene amplification.	0: no staining or weak/incomplete staining (≤ 10% tumor cells)	HER2/CEP17 ratio < 2.0, and the *HER2* gene copy number was < 4.0/cell
Low HER2 expression	HER2 protein expression and gene amplification on the surface of tumor cells are between positive and negative.	2+: > 10% of cells, membrane staining is weak to moderate and completely stained and ISH-negative. 1+: > in 10% of cells, membrane staining was weak to moderate and completely stained and ISH-negative	2+ but ISH-negative: moderate intact or incomplete membrane staining without *HER2* gene amplification (HER2/CEP17 ratio < 2.0 and *HER2* gene copy number < 4.0/cell)
Ultra-low HER2 expression	HER2 protein expression and gene amplification on the surface of tumor cells ranged from low expression to negative.	0: no cell membrane staining or only weak, incomplete membrane staining	Negative: HER2/CEP17 ratio < 2.0, and *HER2* gene copy number < less than 4.0/cell

HER2: human epidermal growth factor receptor 2; CEP17: chromosome enumeration probe; FISH: fluorescence *in situ* hybridization; ISH: *in situ* hybridization; IHC: immunohistochemistry.

## Mechanisms Underlying Trastuzumab’s Antitumor Effects

Trastuzumab’s Fab fragment binds specifically to the HER2 extracellular domain IV (ECD IV) epitope located near the membrane (residues 529–625). This binding irreversibly blocks HER2 function via three distinct mechanisms: 1) ligand-binding blockade by spatial site-blocking, such as hindering the binding of growth factors (e.g., neuromodulators) and preventing growth factors (e.g., neuromodulin) from binding to adjacent receptor structural domains (e.g., HER3-ECD III); 2) conformational interference dimerization, such as locking the extracellular domain of HER2 into a closed conformation and inhibiting the formation of an active heterodimer with HER3/EGFR; and 3) receptor scavenging by inducing trastuzumab-HER2 complex via grid protein-mediated endocytosis and lysosomal degradation pathway, ultimately realizing the complete disruption of the targeted signaling pathway [23]. After conjugation, trastuzumab and HER2 form a trastuzumab-HER2 complex, thereby exerting antitumor effects. This binding is highly selective and targets only HER2-overexpressing tumor cells, reducing damage to surrounding cells, which also leads to a targeted effect while not being able to significantly respond to HER2-underexpressing conditions.

### Inhibition of proliferation signal pathway and induction of cell cycle arrest and apoptosis

Trastuzumab specifically blocks the formation of oncogenic heterodimers between HER2 and HER family members (especially HER3) by binding to HER2 with high affinity to form the trastuzumab-HER2 complex, which in turn inhibits key signaling pathways downstream of HER2. This blockade significantly reduces the activity of the PI3K/AKT pathway (decrease of > 70% in the level of AKT phosphorylation and impaired HER3-mediated recruitment of the PI3K-p85 subunit) and attenuates MAPK/ERK pathway signaling (inhibiting the RAF/MEK/ERK cascade by disrupting Ras-GTP loading and down-regulating cyclin D1 expression). The above dual inhibitions of the pathways ultimately lead to tumor cell cycle arrest in the G1/S phase (CDK4/6 activity inhibition), upregulation of the expression of the pro-apoptotic protein BIM and downregulation of anti-apoptotic protein BCL-2, which synergistically inhibit tumor cell proliferation and weaken their viability [8].

For PI3K/AKT/mTOR pathway, HER2 activation promotes cell survival and anti-apoptosis through the PI3K/AKT pathway, while trastuzumab specifically inhibits PI3K signaling by decreasing PTEN tyrosine phosphorylation and increasing PTEN membrane localization and phosphatase activity [24, 25], thereby inducing cell cycle arrest (such as G1 phase arrest). For MAPK pathway, trastuzumab inhibits the Ras-MAPK pathway and reduces the expression of cell proliferation-related genes (such as c-Fos, c-Jun) [26]. For STAT pathway, trastuzumab inhibits the phosphorylation of STAT3 and reduces the expression of pro-tumor genes (such as Bcl-2 and cyclin D1).

Trastuzumab disengages the inhibitory effect on pro-apoptotic proteins by inhibiting the AKT signaling pathway, specifically by promoting the dephosphorylation of the Ser112/Ser136 site of the BAD protein, thereby releasing its binding to BCL-xL to activate the mitochondrial apoptotic pathway and by disengaging the inhibition of AKT phosphorylation of caspase-9 phosphorylation inhibition by AKT. This mechanism synergistically induces mitochondria-dependent apoptosis. In addition, it effectively inhibits CDK2/cyclin E complex activity by upregulating the cell cycle inhibitory protein p27 and promoting its nuclear translocation, ultimately leading to irreversible cell cycle arrest in the G1 phase [27].

A potentially important mechanism for the immunoreactivity of therapeutic antibodies is antibody-dependent cell-mediated cytotoxicity (ADCC) induced by Fc receptor activation on immune effector cells [28]. Trastuzumab binds to the Fcγ receptor (FcγRIIIa/CD16a) on the surface of immune cells (e.g., natural killer cells, macrophages) through its Fc segment to activate ADCC and antibody-dependent cellular phagocytosis (ADCP), thereby inducing the targeted clearance of tumor cells by immune effector cells. Specifically, the release of perforin/granzyme B from natural killer cells triggers tumor cell lysis, while macrophages synergistically enhance the antitumor effect through phagocytosis and tumor necrosis factor (TNF)-α secretion [29]. At the same time, HER2-overexpressing tumor cells are more easily recognized and cleared by immune cells.

### Inhibition of angiogenesis and metastasis

The extracellular domain of HER2 can be cleaved by matrix metalloproteinases (MMPs) to form soluble p105 protein (sHER2), which promotes tumor metastasis and angiogenesis. Trastuzumab binding to HER2 can prevent the digestion and shedding of its extracellular domain and reduce the cancer-promoting effects of sHER2.

**Figure 2:**
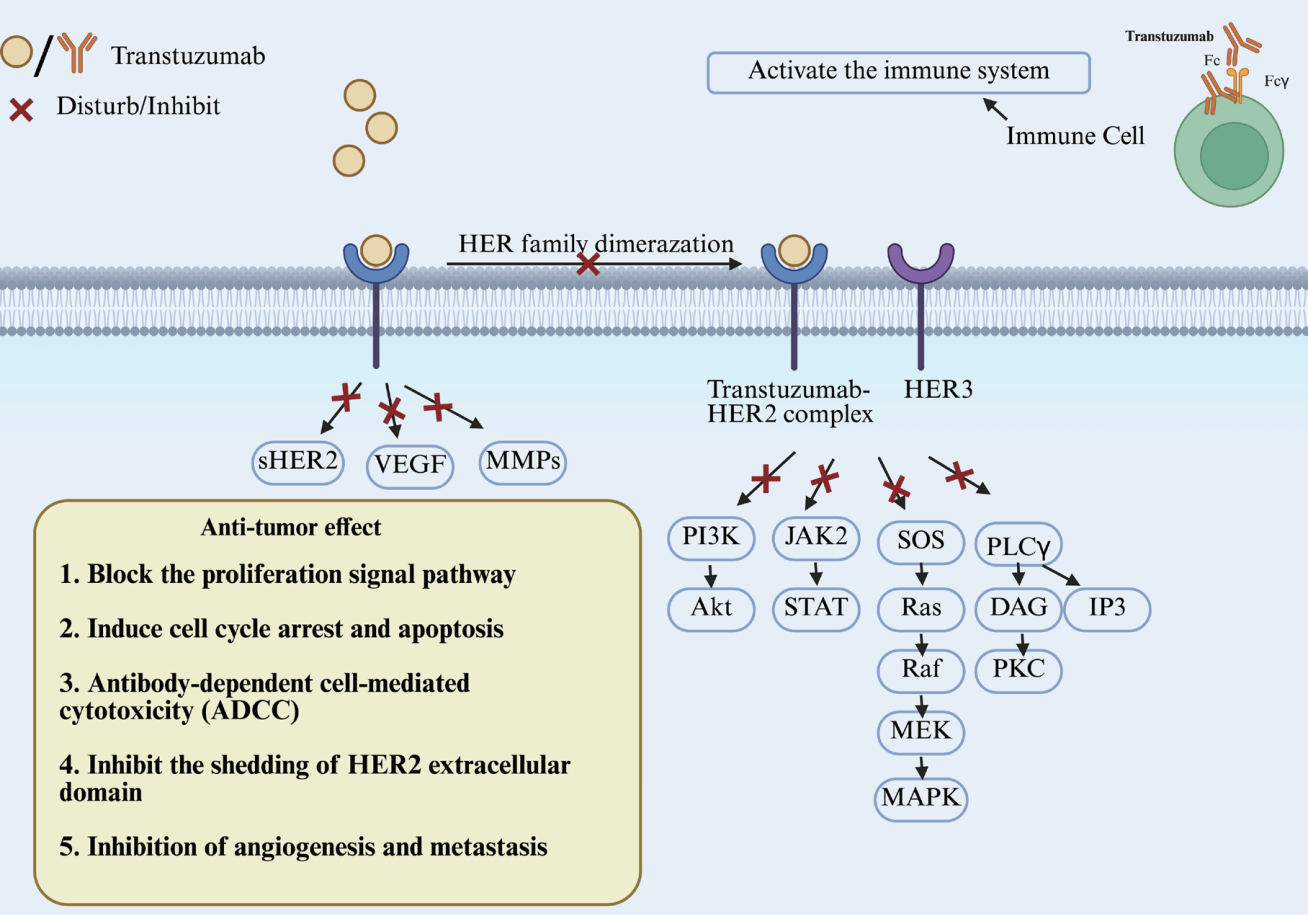
The mechanisms of trastuzumab’s antitumor effect. Trastuzumab binds to human epidermal growth factor receptor 2 (HER2) to form the trastuzumab-HER2 complex, then inhibits the activity of several downstream signaling pathways. VEGF: vascular endothelial growth factor; MMPs: matrix metalloproteinases.

Trastuzumab can also downregulate the expression of vascular endothelial growth factor (VEGF) and MMPs and inhibit tumor angiogenesis, invasion, and metastasis (Fig. 2).

### Limitations of trastuzumab

Although trastuzumab demonstrates significant efficacy against HER2-positive tumors, its effectiveness against HER2-low and HER2-negative tumors remains unsatisfactory. The National Surgical Adjuvant Breast and Bowel Project (NSABP) B-47 phase III randomized trial demonstrated that adding trastuzumab to adjuvant chemotherapy did not improve disease-free survival, distant recurrence-free survival, or overall survival (OS) in high-risk early-stage breast cancer patients with HER2-low expression (IHC 1+ or 2+ and FISH-negative). This outcome confirms the ineffectiveness of conventional anti-HER2 mAb therapy in this population, highlighting a core limitation of current treatment strategies: HER2-low expression cannot yet serve as a predictive biomarker for traditional targeted therapies like trastuzumab, and adjuvant treatment should follow HER2-negative regimens [30]. Although exploratory analyses suggest a potential trend toward reduced OS in the IHC 2+ subgroup receiving trastuzumab, the underlying biological mechanism remains unclear, and these findings warrant cautious interpretation.

In gastric cancer, treatment strategies for HER2-low expression (IHC 1+ or 2+/FISH-negative) face significant limitations. The traditional anti-HER2 mAbs (e.g., trastuzumab) have not demonstrated survival benefits in the tested population, leading to their long-term classification as “HER2-negative” and treatment with conventional chemotherapy [31]. Although ADCs like T-DXd offer new hope for HER2-low gastric cancer, their objective response rates (ORRs) in the DS-8201a in HER2-Expressing Gastric Cancer (DESTINY-Gastric01) trial (26.3% for IHC 2+; 9.5% for IHC 1+) and survival benefits remain significantly lower than their efficacy in breast cancer, highlighting disease biology differences and the limitations of current therapies [7]. Additionally, the spatiotemporal heterogeneity of HER2 expression, insufficient detection standardization (particularly the subjectivity in distinguishing IHC 0 from 1+), and the lack of predictive biomarkers collectively pose core challenges for treatment decision-making [32].

## ADC

ADCs refer to antibody-coupled drugs, an innovative form of drug that combines an mAb with a cytotoxic drug (also known as a “payload” or “warhead”) by means of a chemical linker [33]. ADCs specifically target tumor-associated antigens through the antigen-binding domain of mAbs and utilize a potent cytotoxic payload bridged by a cleavable linker, which selectively releases the toxin after internalization of the antigen-antibody complex and lysosomal degradation, achieving a logarithmic increase in the killing efficacy of tumor cells while significantly reducing off-target toxicity [34]. ADCs combine the targeting of antibodies with the highly effective killing power of cytotoxic drugs to achieve precision treatment of tumor while reducing toxicity to normal tissues.

Since the birth of ADCs, their basic design framework has always been based on the core structure of the trinity: mAbs targeting tumor-associated antigens, potent cytotoxic loads, and bio-cleavable linkers connecting the two. However, the molecular design of each component varies significantly among ADCs, which directly determines the plasma stability, tumor-selective killing efficiency, and clinical therapeutic window of the ADCs by regulating key parameters, such as the drug-antibody ratio (DAR, i.e., the average number of mAb-coupled toxin molecules), which directly determines the plasma stability, tumor-selective killing efficiency, and clinical therapeutic window of ADCs [35]. The efficacy of ADCs therefore depends on the complexity of the interaction of antibodies, linkers and payload components with the tumor and its microenvironment, all of which have important clinical implications [36].

### Comparison of the action characteristics between trastuzumab and trastuzumab-drug conjugate (T-DC)

T-DC refers to an ADC that binds trastuzumab, acting as the mAb component, to a cytotoxic drug through a chemical linker. In addition to the Fab region, the Fc region of the mAb component of the ADC can coordinate ADCC, complement-dependent cytotoxicity, and/or ADCP [23, 37]. At the same time, some ADCs may have a “bystander effect” on neighboring cells, regardless of the compartment in which the payload is released and regardless of the expression of the target antigen [38]. This effect of either drug conjugate kills not only the target antigen-positive cells but also the adjacent antigen-negative cells [39]. This design combines the targeting of trastuzumab with the highly lethal efficacy of cytotoxic drugs and significantly expands its use in HER2-expressing tumors, including HER2-low expressing tumors (Table 2).

**Table 2:** The action Characteristics of Trastuzumab and Trastuzumab-Drug Conjugate (T-DC)

Mechanism	Trastuzumab	T-DC
Primary target	HER2-high expression (IHC 3+ or ISH+)	Low HER2 expression (IHC 1+/2+ or ultra-low expression)
Central	Blocking of signaling pathways, ADCC effects	Targeted delivery of cytotoxins, bystander effects
Dependence	Relies on HER2-high expression and immune microenvironment	Depends on HER2 internalization efficiency and linker stability
Applicable tumor type	Breast cancer, gastric cancer (HER2-positive)	Pan-solid tumors such as breast cancer, gastric cancer, and colorectal cancer
Mechanisms of resistance	HER2 downstream signaling pathway activation and ADCC escape	HER2 expression was lost and the drug efflux pump was upregulated

HER2: human epidermal growth factor receptor 2; IHC: immunohistochemistry; ISH: *in situ* hybridization; ADCC: antibody-dependent cell-mediated cytotoxicity.

### Types of T-DC

Currently, trastuzumab emtansine (T-DM1) and T-DXd are the two currently approved ADCs targeting HER2. T-DXd and the other five T-DCs represent the “second-generation ADCs” that were tested first in HER2-positive breast cancer, then in HER2-low breast cancer and other cancers, showing promising results due to their extraordinary and innovative pharmacokinetic and pharmacodynamic properties [40]. They are novel drugs that are effective in the treatment of solid cancers, and both use trastuzumab as a vehicle to target the HER2 receptor and release cytotoxic drugs through internalization.

T-DM1, the first HER2-targeting ADC approved for advanced and post-neoadjuvant breast cancer, has a humanized immunoglobulin (Ig)G1 mAb covalently coupled to the microtubule inhibitor maytansinoid DM1 via a non-cleavable thioether linker (4-(N-maleimidomethyl)cyclohexane-1-carboxylate, MCC), with an average loading of 3.5 DM1 molecules per antibody. The coupling is achieved via an amide bond formation reaction between the ε-amino group of trastuzumab heavy chain lysine residues (K246/K288/K317 sites) and the MCC-NHS ester, resulting in a highly stable complex (plasma half-life of approximately 4 days) that is designed to balance the efficiency of targeted delivery with the risk of systemic toxicity [41].

However, due to the non-cleavage of its linker, T-DM1 can only exert a limited bystander effect, so it has no significant effect on HER2-low tumors. And brain metastases occur in about 40% of advanced patients with HER2+ breast cancer, and these patients usually have a poor prognosis [34]. It has been shown that T-DM1 has relatively limited efficacy in treating complicating brain metastases [35]. Moreover, the use of T-DM1 has been reported to cause pulmonary toxicity, hepatotoxicity, and skin radiation toxicity, with thrombocytopenia being another notable complication [42–44].

T-DXd, a next-generation HER2-targeted ADC, received accelerated approval from the United States Food and Drug Administration (FDA) in 2019 for patients with unresectable or metastatic HER2-positive breast cancer who has failed anti-HER2 therapy [45]. As a novel HER2-targeted ADC, T-DXd consists of a humanized mAb attached to a potent topoisomerase I inhibitor payload via a cleavable peptidyl linker. Anti-HER2 antibody is a humanized monoclonal IgG1 reference to the same produced amino acid sequence as trastuzumab. The linker is stabilized in plasma and upregulated by lysosomal selective cleavage of catechin in cancer cells [46]. The synergistic combination of a high DAR (approximately 8) and a cleavable linker enhances the targeted delivery of the topoisomerase I inhibitor to tumor cells. This design, coupled with its optimized stability in plasma, reduces systemic exposure and consequently lowers the risk of toxicity such as neutropenia. The core of its innovation lies in the potent bystander effect, mediated by loaded membrane permeability (logP = 1.8). Free DXd, released by lysosomal cleavage, can freely penetrate the target cell membrane and diffuse into neighboring HER2-negative/low-expressing tumor cells to achieve cross-target killing by inducing DNA double-strand breaks to achieve cross-target killing and overcome tumor heterogeneity [47]. High DARs and cytotoxic side-effects may target tumors with heterogeneous HER2 expression, such as gastric cancer tumors [48].

Compared with the previous trastuzumab ADC, T-DXd has a more significant toxic effect on HER2-low tumor cells. This also provides a new idea for the application of trastuzumab in HER2-low tumors. Meanwhile, according to the results of the randomized phase III DS-8201a Versus T-DM1 for HER2-Positive, Unresectable and/or Metastatic Breast Cancer Previously Treated With Trastuzumab and Taxane (DESTINY-Breast03) trial, T-DXd demonstrated meaningful intracranial efficacy and clinical benefit in OS and had an acceptable and manageable safety profile in patients with HER2-positive metastatic breast cancer (treated/stable and untreated/active brain metastases) [49, 50]. However, although T-DXd has a high therapeutic effect, it can also cause a variety of adverse events, including interstitial lung disease (ILD) and gastrointestinal toxicity [51, 52]. Therefore, in the process of ensuring patient safety outcomes, it is necessary to optimize the strategy of careful monitoring [53].

It is clear that T-DXd has significant advantages over the T-DM1 in all aspects (Table 3). For linker stability, the non-cleavable linker of T-DM1 relies on the complete degradation of lysosomes to release the load, while the cleavable linker of T-DXd can quickly release the load in the tumor microenvironment. For payload type, DM1 of T-DM1 inhibits microtubule polymerization, while T-DXd consists of trastuzumab, cleavable tetrapeptide linker, and highly active topoisomerase I inhibitor (DXd), which kills cells through DNA damage mechanisms. For DAR, T-DXd carries eight DXd molecules per antibody, and the DAR value (8 vs. 3.5) improves the drug delivery efficiency of a single treatment and significantly improves the killing efficiency. After the linker of T-DXd is lysed in the tumor microenvironment, the released DXd is membrane-permeable and can penetrate the cell membrane to kill adjacent HER2-low or HER2-negative tumor cells. This mechanism enables T-DXd to exert significant efficacy in HER2-low tumors. However, T-DM1 has a weak bystander effect due to its non-cleavable linker limitation, which mainly relies on the targeted effect of HER2-high expression cells for precise killing.

**Table 3:** Characteristics, Mechanism of Action and Clinical Application of the Two Types of Trastuzumab-Drug Conjugate (T-DC)

Characteristic	Trastuzumab emtansine (T-DM1)	Trastuzumab deruxtecan (T-DXd)
Connector	Non-cleavable	Cleavable
Load	Microtubule inhibitor	Topoisomerase I inhibitor
Drug-antibody ratio	3.5	8
Bystander effect	Limited	Significant
Indications	HER2-positive breast cancer	HER2-positive/-low breast cancer, gastric cancer
Efficacy of brain metastases	Limited	Significant
Major toxicity	Thrombocytopenia, hepatotoxicity	Interstitial lung disease, gastrointestinal toxicity

HER2: human epidermal growth factor receptor 2.

The ADC formats of trastuzumab (T-DM1 and T-DXd) significantly enhance the therapeutic efficacy of HER2-expressing tumors by combining targeting and cytotoxicity. T-DXd has become a breakthrough drug for the treatment of HER2-low tumors due to its high bystander effect and high DAR. Currently, T-DXd has been approved for the treatment of HER2-expressing breast and gastric cancers and HER2-mutated non-small cell lung cancers, although its efficacy in other HER2-expressing solid tumors remains limited [54].

T-DXd demonstrated clinically meaningful efficacy across multiple HER2-positive (IHC 2+/3+) advanced solid tumors in pretreated patients, achieving an ORR of 37.1%, confirming its potential for “cross-cancer” treatment [54]. DESTINY-Breast04 established T-DXd as a new standard of care for previously treated HER2-low metastatic breast cancer, significantly extending progression-free survival (PFS) and OS compared to chemotherapy [55]. DESTINY-Breast06 further extended this benefit to patients who developed resistance to endocrine therapy and had not received prior chemotherapy (Table 4) [54–58], including those with extremely low HER2 expression (IHC 0 with no membrane staining), significantly broadening the patient population eligible for treatment [56].

**Table 4:** Details of Several T-DXd trials

Trial name	Stage	Crowd	Interventions	Key efficacy endpoints	References
DESTINY-PanTumor02	II	Patients with previously treated, locally advanced or metastatic HER2-expressing (IHC 3+/2+) solid tumors (including endometrium, cervix, ovary, bladder, biliary tract, pancreas and other cancers)	T-DXd	For all patients: confirmed ORR: 37.1% (95% CI, 31.3–43.2); median PFS: 6.9 months (95% CI, 5.6–8.0); median OS: 13.4 months (95% CI, 11.9–15.5)	[54]
				For centrally confirmed IHC 3+ patients: confirmed ORR: 61.3% (95% CI, 49.4–72.4); median PFS: 11.9 months (95% CI, 8.2–13.0); median OS: 21.1 months (95% CI, 15.3–29.6)	
DESTINY-Breast04	III	Patients with prior chemotherapy, unresectable or metastatic HER2-low expression (IHC 1+ or IHC 2+/ISH–) breast cancer	T-DXd vs. chemotherapy of doctor’s choice (TPC)	Median PFS: 10.1 vs. 5.4 months (T-DXd vs. TPC); median OS 23.9 vs. 17.5 months (T-DXd vs. TPC)	[55]
DESTINY-Breast06	III	Patients with HR-positive, HER2-low, or very low-expressing mBC who have progressed after at least first-line endocrine therapy and have not received metastatic chemotherapy	T-DXd vs. chemotherapy of doctor’s choice (TPC: capecitabine/albumin paclitaxel/paclitaxel)	For HER2-low expression population: median PFS: 13.2 vs. 8.1 months (T-DXd vs. TPC).	[56]
				For ITT population, including very low HER2 expression): median PFS: 13.2 vs. 8.1 months.	
DESTINY-Gastric02	II	Patients with HER2-positive advanced gastric or gastroesophageal junction cancer with disease progression on or after a trastuzumab-containing regimen	T-DXd	Confirmed ORR: 41·8% (95% CI, 30·8–53·4); median PFS: 5.6 months (95% CI, 4.2–8.3); median OS: 12.1 months (95% CI, 9.4–15.4); median DoR: 8.1 months (95% CI, 5.9–NE); DCR: 81.0% (64/79; 95% CI, 70.6–89.0); median TTR: 1.4 months (95% CI, 1.4–2.7); CR: 5.1%; PR: 36.7%.	[57]
					
DAISY	II	Patients with mBC stratified by HER2 expression level into three cohorts: HER2-overexpressing, HER2-low, and HER2-non-expressing	T-DXd	Primary endpoint (confirmed ORR): cohort 1, 70.6% (95%, CI 58.3–81); cohort 2, 37.5% (95% CI, 26.4–49.7); cohort 3, 29.7% (95% CI, 15.9–47).	[58]
				Secondary endpoints (median PFS): cohort 1, 11.1 months (95% CI, 8.5–14.4); cohort 2, 6.7 months (95% CI, 4.4–8.3); cohort 3, 4.2 months (95% CI, 2.0–5.7)	
				Median OS (not fully mature at data cutoff): cohort 1 and cohort 2, not reached; cohort 3, 11.6 months.	
				CBR: cohort 1, 85.3%; cohort 2, 56.9%; cohort 3, 35.1%.	
				DoR: cohort 1, 9.7 months; cohort 2, 7.6 months; cohort 3, 6.8 months.	

mBC: metastatic breast cancer; TPC: treatment of the physician’s choice; OS: overall survival; CI: confidence interval; CR: complete response; DCR: disease control rate; DoR: duration of response; ORR: objective response rate; PFS: progression-free survival; PR: partial response; CR: complete response; TTR: time to response; CBR: clinical benefit rate; HER2: human epidermal growth factor receptor 2; T-DXd: trastuzumab deruxtecan; ITT: intent-to-treat; IHC: immunohistochemistry.

### Adverse effects of T-DC

ILD represents a key toxicity of concern during the clinical application of anti-HER2 ADCs. Current clinical evidence indicates an overall incidence of anti-HER2 ADC-associated ILD at 4.4% (all grades) and 0.5% (≥ grade 3) [57]. Pooled data from multiple clinical studies show that the incidence of T-DXd-associated ILD/pneumonitis varies across tumor types and trials, ranging broadly from 11% to 15.8%. Most events were grade 1–2, with severe (≥ grade 3) events occurring at approximately 3.5% and fatal (grade 5) events at 0.1–1.0% [58]. In the DESTINY-Lung01 trial, the incidence of ILD was notably higher (26.4%) among patients with *HER2*-mutated non-small cell lung cancer [59]. The median time to onset of ILD/pneumonitis was approximately 5.5 months, with the vast majority (97%) occurring within the first 12 months of treatment initiation and no apparent evidence of cumulative toxicity. Risk factors may include treatment in Japan, higher doses (e.g., 6.4 mg/kg), lower baseline oxygen saturation, moderate to severe renal impairment, and specific pulmonary comorbidities [58].

The severity of this toxicity is graded according to the Common Terminology Criteria for Adverse Events (CTCAE) established by the National Cancer Institute. Grade 1 is asymptomatic with imaging findings only; grade 2 and above present with symptoms such as dyspnea, cough, or fever; severe cases may progress to diffuse alveolar damage, posing a life-threatening risk.

Clinically, ILD events predominantly occur early in treatment, with a median onset time of 86 days. Over half of cases developed within 3 months, with T-DXd showing a significantly earlier median onset (44 days) compared to T-DM1 (129 days). According to CTCAE grading, ≥ grade 3 ILD constitutes a serious adverse event, manifesting as progressive dyspnea, decreased oxygenation, or even respiratory failure, with a mortality rate reaching 18.8% [57]. Therefore, it warrants prioritization in clinical monitoring and management.

For ILD monitoring, consensus recommends a proactive strategy: baseline pulmonary function and chest imaging should be assessed prior to treatment; during therapy (especially within the first 3–6 months), patients require close follow-up for respiratory symptoms and regular high-resolution computed tomography (CT) scans; monitoring frequency should be intensified for high-risk populations (e.g., Asian patients, those receiving T-DXd). Management follows a tiered approach: grade 1–2 ILD requires treatment interruption and consideration of corticosteroids until symptomatic and radiographic improvement; grade ≥ 3 ILD necessitates permanent discontinuation of the relevant ADC, immediate initiation of high-dose corticosteroids, and, if necessary, respiratory support and multidisciplinary team intervention [55].

In the DESTINY-PanTumor02 phase II trial, gastrointestinal toxicity was the most common adverse reaction observed with T-DXd treatment in HER2-expressing solid tumors, with incidence rates of nausea (55.1%), diarrhea (25.8%), and vomiting (24.7%) [54]. Most events were grade 1–2, while grade ≥ 3 events were relatively uncommon [54].

Monitoring requires symptom assessment prior to each treatment cycle, graded according to CTCAE v5.0. Management strategies focus on prevention and symptomatic relief: prophylactic use of 5-HT3 receptor antagonists is recommended; loperamide may be administered for diarrhea with attention to hydration; for persistent or ≥ grade 3 gastrointestinal toxicity, treatment interruption or dose reduction should be considered [54, 58].

T-DM1-associated hepatotoxicity primarily manifests as elevated serum transaminases. In the EMILIA phase III trial, the incidence rates of any-grade aspartate aminotransferase (AST) and alanine aminotransferase (ALT) elevations were 22.4% and 16.9%, respectively, with lower incidence rates for grade ≥ 3 events (4.3% and 2.9%, respectively). No cases met the hepatitis C virus criteria for drug-induced liver injury, indicating a low risk of severe hepatic injury [60]. For clinical management, baseline and periodic monitoring of liver enzymes during treatment is recommended. If grade ≥ 3 elevations occur, treatment should be suspended and resumed at a reduced dose once levels return to grade ≤ 1. Prophylactic hepatoprotective agents are generally unnecessary. Overall, this toxicity is manageable, and patient safety can be ensured through monitoring and dose adjustments.

Thrombocytopenia is one of the primary hematologic toxicities observed with T-DM1 clinical use. The pivotal phase III EMILIA study demonstrated a 48% incidence of grade ≥ 3 adverse events in the T-DM1 group, with thrombocytopenia being a common laboratory abnormality, particularly prevalent during the initial treatment period. This toxicity is predominantly grade 1–2, but some patients may experience grade 3–4 decreases, warranting clinical attention. Monitoring recommendations include routine complete blood counts (CBCs) prior to each treatment cycle, with additional CBCs as clinically indicated during therapy. Management strategies include considering dose delay or adjustment (e.g., reducing to 3.0 mg/kg) for moderate-to-severe thrombocytopenia (< 50 × 10^9^/L). Platelet transfusions or thrombopoietic agents may be administered as needed. Continuous bleeding risk assessment throughout treatment is essential to achieve an individualized balance between efficacy and safety.

## Combination Chemotherapy and Combination Immunotherapy With Trastuzumab

### Combination chemotherapy with trastuzumab

During treatment, trastuzumab specifically binds to the HER2 receptor, inhibits downstream signaling pathways, blocks tumor cell proliferation, and induces ADCC. Chemotherapy drugs (e.g., paclitaxel, docetaxel, carboplatin, and fluorouracil) directly kill rapidly dividing tumor cells and synergistically enhance tumor cell death with trastuzumab. Chemotherapy may disrupt the tumor microenvironment, enhance the penetration and target of trastuzumab, and reduce drug resistance due to monotherapy. Several adjuvant trials have shown that the addition of trastuzumab to chemotherapy reduces the risk of recurrence and death in women with early-stage breast cancer who were overexpressed or genetically amplified with HER2 [49].

The ToGA phase III clinical trial demonstrated that for patients with HER2-positive advanced gastric cancer or gastroesophageal junction cancer, trastuzumab combined with chemotherapy significantly improved OS compared to chemotherapy alone (13.8 months vs. 11.1 months, (hazard ratio) HR = 0.74, P = 0.0046), with comparable safety profiles [31]. Subgroup analysis revealed greater benefit in patients with high HER2 expression (IHC 3+ or IHC 2+ and FISH-positive) (median OS 16.0 months vs. 11.8 months). This study supports trastuzumab plus chemotherapy as the standard first-line treatment for HER2-positive advanced gastric cancer [31].

The NOAH trial demonstrated that, in patients with HER2-positive locally advanced or inflammatory breast cancer, the adding of trastuzumab (administered from the preoperative through postoperative period for a total of 1 year) to neoadjuvant chemotherapy significantly improved 3-year event-free survival (71% vs. 56%, HR = 0.59, P = 0.013) and markedly increased pathological complete response rates (43% vs. 22%, P = 0.0007), while demonstrating favorable cardiac safety (only 2% symptomatic heart failure incidence). These findings support the use of trastuzumab-containing neoadjuvant regimens in this patient population [61].

Currently, trastuzumab combined with chemotherapy shows limited efficacy for HER2-low tumors and is not clinically recommended. However, the emergence of T-DCs has overcome this limitation. The combination of T-DCs with chemotherapy, specifically T-DCs combined with chemotherapy, holds significant clinical potential.

Given the large intra-heterogeneity between HER2-low and HER2-positive tumors, the combination with multiple chemotherapies may be beneficial for HER2-low tumors. Currently, some existing studies have shown that T-DXd has great potential in combination with nivolumab and capecitabine and oxaliplatin (CAPOX) for the treatment of patients with HER2-low gastroesophageal adenocarcinoma. In addition, preclinical studies in mouse models have shown that T-DXd activates dendritic cells and enhances major histocompatibility complex class I (MHC-I) expression in tumor cells, suggesting a synergistic effect with anti-programmed death-1 (PD-1) therapy [51]. At the same time, the combination of first-line T-DXd with rilvegostomig—a PD-1/T-cell immune receptor with Ig and immunoreceptor tyrosine-based inhibitory motif (ITIM) domains (TIGIT) bispecific antibodies—and fluoropyrimidine (FP) was investigated in patients with HER2-positive or HER2-negative hypogastric cancer [52]. In some trials, T-DXd in combination with anastrozole or fulvestrant can also be used to treat patients with HER2-low hormone receptor (HR)-positive advanced/metastatic breast cancer [53].

All of the above shows that the combination of T-DC with chemotherapy has considerable prospects in the treatment of HER2-low tumors.

### Combined immunotherapy with trastuzumab

Trastuzumab combined with immunotherapy is a strategy that fights cancer by synergistically activating or enhancing the immune system. It involves combining trastuzumab with other immunotherapy approaches, such as immune checkpoint inhibitors, cytokine therapy, chimeric antigen receptor T-cell (CAR-T) cell therapy, and others, to overcome the tumor’s immunosuppressive microenvironment and enhance antitumor efficacy [62].

Currently, the combination of trastuzumab with immune checkpoint inhibitors shows great promise in treating HER2-positive cancers. During treatment, immunologically activated trastuzumab binds to HER2 receptors, recruiting natural killer cells, macrophages, and other immune cells to kill tumors, release tumor antigens, and activate T-cell immune responses. Immune checkpoint inhibitors alleviate PD-1/programmed cell death ligand 1 (PD-L1) signaling suppression in T cells, enhancing immune-mediated killing activity within the tumor microenvironment. Regulation of HER2 signaling within an immunosuppressive microenvironment may upregulate PD-L1 expression in tumor cells, facilitating immune escape. Combination therapy blocks the PD-L1 pathway, reverses immunosuppression, and promotes T-cell infiltration. The synergistic effect of targeted therapy reduces tumor burden, while immunotherapy eliminates residual disease and lowers recurrence risk, particularly in metastatic or refractory tumors.

The phase III KEYNOTE-811 study confirmed that adding pembrolizumab to trastuzumab plus chemotherapy significantly improved ORR, PFS, and OS trends, with greater benefits observed in patients with PD-L1 combined positive score (CPS) ≥ 1. This combination has been established as a new standard first-line regimen [63]. Combination therapy with nivolumab, trastuzumab, and chemotherapy (FOLFOX) demonstrated superior efficacy compared to historical controls in phase II studies such as AIO INTEGA, providing an effective alternative regimen [64].

Some experiments have shown that low-pan-HER inhibitors induce superior antitumor effects compared to single-agent inhibitors. For example, the combination of T-DXd and irreversible pan-HER tyrosine kinase inhibitor (pan-HERTKI) afatinib can promote the benefit of combination therapy in HER2-low expression gastric and lung tumors [55]. In the HR-negative/HER2-low phase III DESTINY-Breast04 trial, the investigators conducted a preclinical study of T-DXd combined with DNA damage response pathway inhibitors in a xenograft model of HER2-low/HR-negative breast cancer patients, and the results showed that it had great potential for the treatment of HER2-low/HR-negative breast cancer patients [56].

The above results indicate that the combination of T-DC immunotherapy also plays a non-negligible role in the treatment of HER2-low tumors.

## Expectation

Standardization and precision in HER2 testing represent core challenges for future clinical applications, with the primary task being to address the subjectivity and reproducibility issues in interpreting IHC 0 and 1+ results. Current IHC methods face significant difficulties in distinguishing low-level expression, including pre-processing factors (such as fixation and antigen retrieval), antibody clone selection, variations in staining protocols, and subjective interpretation differences among observers. These factors collectively contribute to inconsistencies in reporting low HER2 expression, particularly when differentiating between IHC 0 (including ultra-low expression) and 1+ [65, 66]. Furthermore, intratumoral HER2 expression heterogeneity further complicates accurate assessment [67]. To overcome these limitations, future efforts should focus on developing more objective, quantitative detection technologies. For instance, standardized assays like the VENTANA anti-HER2/neu (4B5) antibody have been established as companion diagnostics [68]. Meanwhile, methods developed by Moutafi et al, combining quantitative immunofluorescence with mass spectrometry, enable direct measurement of absolute HER2 protein levels (attomoles/mm^2^) on tissue sections, providing a more precise quantitative cutoff for HER2-low tumors without gene amplification [69]. Mass spectrometry techniques (e.g., immunoaffinity enrichment coupled with targeted mass spectrometry) have demonstrated their capability for quantifying HER2 protein in formalin-fixed paraffin-embedded (FFPE) samples, showing strong correlations with IHC/ISH status and clinical outcomes, thereby providing reliable tools for precise quantification [70, 71]. Concurrently, artificial intelligence (AI) and digital pathology image analysis demonstrate significant potential. AI-assisted systems can provide more reliable and reproducible scoring by analyzing stained images and calculating membrane staining intensity. Studies have shown their ability to improve the accuracy of HER2 0 and 1+ interpretation, particularly when handling heterogeneous samples [72–74]. Integrating these innovative digital and molecular technologies, supported by rigorous standardized operating procedures, continuous quality control, and pathologist training, will fundamentally enhance the precision and consistency of HER2 low-expression detection. This ensures that appropriate patients are accurately matched to novel targeted therapies [65, 75].

Trastuzumab is a mAb targeting HER2 that exerts significant therapeutic effects in HER2-positive breast and gastric cancers through mechanisms such as signaling pathway blockade and ADCC induction, but its efficacy is limited in tumors with low HER2 expression. ADCs, such as T-DM1 and T-DXd, combine targeting with cytotoxicity. Particularly, T-DXd significantly expands the therapeutic prospects for HER2-low tumors through its high DAR, cleavable linker, and potent bystander effect. Concurrently, novel drug modalities like bispecific antibodies and antibody-oligonucleotide conjugates (AOCs) are expanding the targeted therapy arsenal, establishing new paradigms for precision delivery of diverse therapeutic modalities (e.g., oligonucleotides, cytotoxins) [76].

Regarding combination strategies, trastuzumab synergistically enhances antitumor effects when paired with chemotherapy or immunotherapies (e.g., immune checkpoint inhibitors). However, traditional combination regimens remain limited for HER2-low tumors, whereas T-DC-based combination therapies show promise. Future exploration of combination strategies will transcend simple drug stacking, focusing instead on rationally designed synergistic combinations based on mechanism of action. Examples include ADC combinations with immune checkpoint inhibitors, poly (ADP-ribose) polymerase (PARP) inhibitors, or novel cell therapies, aiming to simultaneously overcome tumor microenvironment suppression, activate systemic immune responses, and eliminate persistent tumor cells.

## Data Availability

The authors declare that data supporting the findings of this study are available within the article.
